# Supplementary material for: A Global Screen for Assembly State Changes of the Mitotic Proteome by SEC-SWATH-MS
Source: Cell Syst. 2020 Feb 26;10(2):133–155.e6. doi: 10.1016/j.cels.2020.01.001 (PMC7042714; doi:10.1016/j.cels.2020.01.001)

Q14527 | HLTF_HUMAN | HLTF HIP116A RNF80 SMARCA3 SNF2L3 ZBU1
Monomer MW [kDa]: 113.929 Monomer expected elution fraction: 39

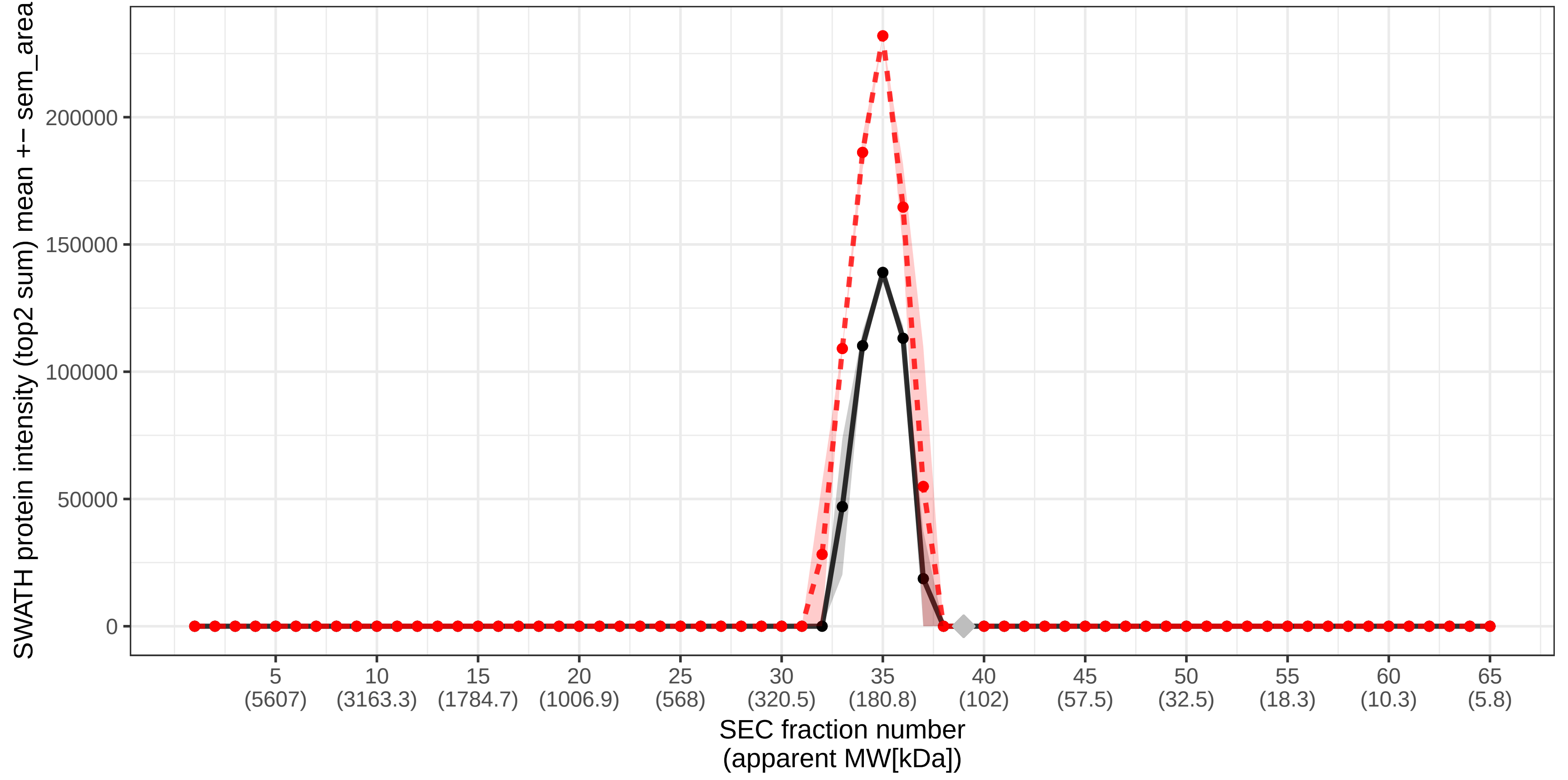

Supplement: Data S1. SEC-SWATH-MS Protein Chromatograms, Related to Figure 1 [file mmc6.zip › SECchrom_Q14527_HLTF_HUMAN_HLTF_HIP116A_RNF80_SMARCA3_SNF2L.pdf]
